# Highly efficient hole injection from Au electrode to fullerene-doped triphenylamine derivative layer

**DOI:** 10.1038/s41598-022-10983-6

**Published:** 2022-05-04

**Authors:** Shofu Matsuda, Chikara Itagaki, Kyoya Tatsuguchi, Masamichi Ito, Hiroto Sasaki, Minoru Umeda

**Affiliations:** Department of Materials Science and Technology, Graduate School of Engineering, Nagaoka University of Technology, 1603-1 Kamitomioka, Nagaoka, Niigata 940-2188 Japan

**Keywords:** Electrochemistry, Electron transfer

## Abstract

Triphenylamine derivatives are superior hole-transport materials. For their application to high-functional organic semiconductor devices, efficient hole injection at the electrode/triphenylamine derivative interface is required. Herein, we report the design and evaluation of a Au/fullerene-doped α-phenyl-4′-[(4-methoxyphenyl)phenylamino]stilbene (TPA) buffer layer/TPA/Au layered device. It exhibits rectification conductivity, indicating that hole injection occurs more easily at the Au/fullerene-doped TPA interface than at the Au/TPA interface. The Richardson-Schottky analysis of the device reveals that the hole injection barrier (*Φ*_B_) at the Au/fullerene-doped TPA interface decreases to 0.021 eV upon using C_70_ as a dopant, and *Φ*_B_ of Au/TPA is as large as 0.37 eV. The reduced *Φ*_B_ of 0.021 eV satisfies the condition for ohmic contact at room temperature (*Φ*_B_ $\le$ 0.025 eV). Notably, C_70_ doping has a higher barrier-reduction effect than C_60_ doping. Furthermore, a noteworthy hole-injection mechanism, in which the ion–dipole interaction between TPA and fullerenes plays an important role in reducing the barrier height, is considered based on cyclic voltammetry. These results should facilitate the design of an electrode/organic semiconductor interface for realizing low-voltage driven organic devices.

## Introduction

Semiconductor devices have attracted considerable attention in recent years. In particular, organic semiconductors are being used in devices including organic photovoltaics (OPVs)^1–3^, organic light-emitting diodes (OLEDs)^3–6^, and organic field-effect transistors^2,7–13^ including for fabricating biosensors and radio frequency identification (RFID) tags. Organic semiconductor devices afford advantages such as a low-temperature fabrication process, low cost, and high flexibility. However, they have low carrier mobility and high carrier injection barriers compared with inorganic semiconductor devices. To overcome this disadvantage, organic semiconductors prepared by single crystallization and having a mobility of 1 cm^2^ V^−1^ s^−1^ or more, which exceeds that of amorphous silicon, have been fabricated recently^14,15^. Regarding carrier injection, the formation of the barrier depends on the energetic alignment of an electrode and an organic semiconductor^16^. Hence, the interface modifications such as the introduction of hole injection layers (i.e., buffer layers), electrode surface modification, and dopant implantation has been conventionally performed to reduce the barrier height by generating new energetic interface states^3,5,15,17–24^.

Triphenylamine derivatives are well recognized as a superior hole-transport material. Stacked photoelectric conversion devices with triphenylamine derivative as a hole transport layer are used in the organic photoreceptors of laser printers^25,26^. Triphenylamine derivative single crystals produced by the solution method^27–29^ exhibit excellent conductive properties, and their hole-transport activation energy is equivalent to that of pentacene^30^ and rubrene^21^ single crystals. Furthermore, stable and energetically favorable hole transport has been achieved via the first oxidation state of triphenylamine derivative^31^. Therefore, triphenylamine derivatives are attracting attention for application to highly functional organic devices. In this regard, efficient hole injection is required at the electrode/triphenylamine derivative interface. In our previous study, we reduced the interfacial energy barrier of a Au/triphenylamine derivative layered device to 0.06 eV by inserting a C_60_-doped triphenylamine derivative buffer layer at the interface^32^. However, further investigations need to be conducted to achieve lower barrier heights (i.e., ohmic contact) and to clarify the barrier height reduction mechanism.

**Figure 1:**
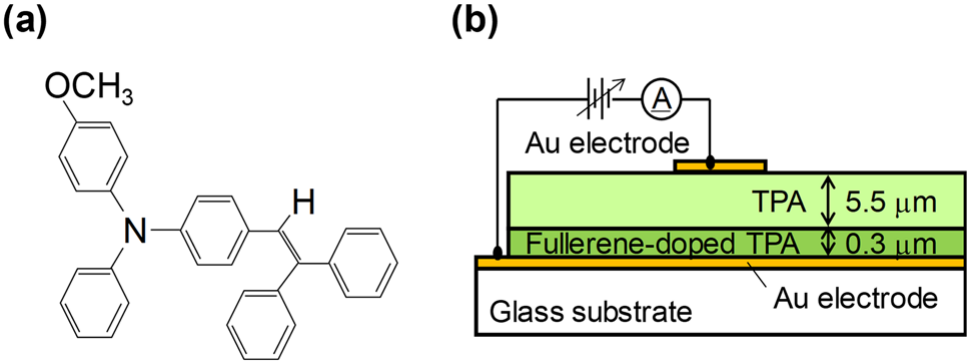
(**a**) Molecular structure of α-phenyl-4′-[(4-methoxyphenyl)phenylamino]stilbene (TPA) and (**b**) schematic cross-section of the Au/fullerene-doped TPA/TPA/Au layered device.

In this study, we report a Au/triphenylamine derivative layered device that has an ohmic contact with hole injection (*Φ*_B_ = *kT* ≤ 0.025 eV, where *Φ*_B_ is the hole injection barrier, *k* is the Boltzmann constant, and *T* is the temperature) at room temperature (298 K) and a novel hole-injection mechanism. We used an additional hole injection layer with a mixture of fullerene and triphenylamine derivative. Further, we used not only C_60_ but also C_70_ as a fullerene, and we used α-phenyl-4′-[(4-methoxyphenyl)phenylamino]stilbene (Fig. 1a) as a triphenylamine derivative and call it TPA. Fullerenes are known to be famous electron transport materials for OPVs and OLEDs^33–35^, but no study has yet applied C_70_ to the hole injection layer. A Au/fullerene-doped TPA/TPA/Au layered device was prepared, and its rectification characteristics were evaluated. In particular, the hole injection property at the Au/fullerene-doped TPA interface was quantitatively evaluated. Further, we conducted cyclic voltammetry to clarify the hole-injection mechanism at this interface.

## Results and discussion

### Hole-injection property of fabricated device

**Figure 2:**
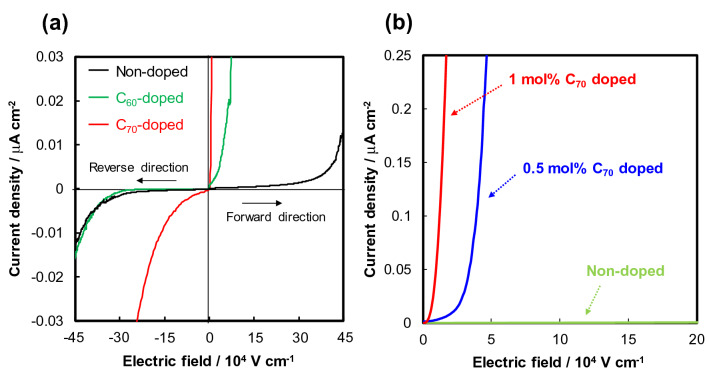
*J*-*E* characteristics at room temperature of the Au/fullerene-doped TPA/TPA/Au layered devices (**a**) in forward and reverse directions when 1 mol% C_60_ and C_70_ was doped, and (**b**) as a function of C_70_ doping amount in forward direction.

Figure 1b shows a schematic of the fullerene-doped TPA dual-layer device fabricated in this study. Hole injection from the bottom and the top Au electrode was defined as the forward and reverse direction, respectively. Figure 2a shows the current density–electric field (*J*-*E*) properties of the Au/C_60_ and C_70_-doped TPA/TPA/Au layered devices. Symmetrical *J*-*E* characteristics were observed in the forward and reverse directions for the non-doped (Au/TPA/Au) device, indicating that the energy barriers for hole injection at both Au/TPA interfaces were equal. By contrast, the threshold electric field of the C_60_-doped device was confirmed to be lowered only in the forward direction; further, that of the C_70_-doped device was drastically suppressed in the forward direction. These results demonstrated that hole injection at the Au/fullerene-doped TPA interface occurs easily compared to that at the Au/TPA interface. It should be noted that the threshold electric fields of the non-doped and the C_60_-doped devices in the reverse direction are almost the same. Therefore, we successfully developed a device with a rectifying property by inserting C_60_- and C_70_-doped TPA buffer layers. This observed rectification conductivity is very useful for organic semiconductor device applications such as RFID tags. In the reverse direction in Fig. 2a, the reason why a higher current was observed in the C_70_-doped device than in the C_60_-doped device remains unclear. Figure 2b shows the dependence of the *J*-*E* characteristics in the forward direction on the C_70_ doping amount (0, 0.5, and 1 mol%) in TPA. A higher current density was achieved at a lower electric field as the C_70_ doping concentration increased. Therefore, fullerene doping induced efficient hole injection.

**Figure 3:**
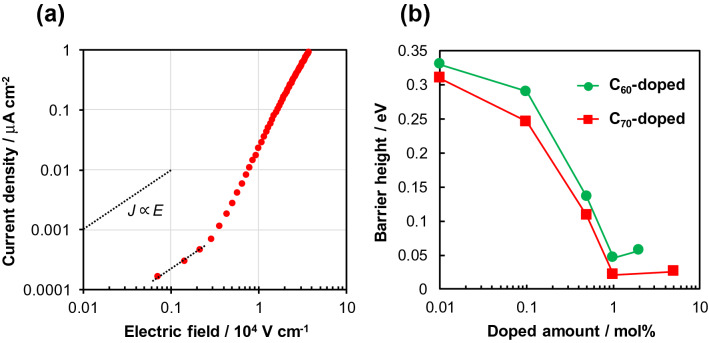
(**a**) *J*-*E* characteristics at 24.6 °C of the Au/1 mol% C_70_-doped TPA/TPA/Au layered device plotted on a log–log scale. The black dotted line indicates the slope of the *J* $\propto$ *E* relationship. (**b**) Dependence of barrier height at the Au/fullerene-doped TPA interface on the fullerene doping amount.

To consider the influence of fullerene doping on hole injection in detail, the *J*-*E* property of the Au/1 mol% C_70_-doped TPA/TPA/Au layered device was plotted on a logarithmic scale, as shown in Fig. 3a. Generally, the *J*-*E* characteristics of organic semiconductors consist of three types of current: (1) Schottky current (*J* ∝ *E*^0.5^), (2) ohmic current (*J* ∝ *E*), and (3) space charge limited current (*J* ∝ *E*^x^ (2 $\le$ x)). Significantly, Fig. 3a showed that the 1 mol% C_70_-doped device exhibited an ohmic-type current response immediately after an electric field was applied, indicating ohmic contact with hole injection at the Au/C_70_-doped TPA interface at room temperature. In other words, no Schottky current was observed in the device. Subsequently, hole transport became a rate-determining process from around 0.2 × 10^4^ V cm^−1^, and a space charge limited current was observed in Fig. 3a. It should be noted that *J* ∝ *E*^0.5^ relationships were observed in the log*J*-log*E* characteristics of the Au/1 mol% C_70_-doped TPA/TPA/Au device under the measurement temperature conditions of 4.5 °C and − 22.3 °C as shown in Supplementary Fig. S1.

We quantitatively evaluated the energy barrier height for hole injection at the Au/fullerene-doped TPA interface through a Richardson-Schottky plot analysis^32,36^. The hole-injection barrier height (*Φ*_B_) was obtained using the following equation:

1 $$\mathrm{ln}\left(J/{T}^{2}\right)=\mathrm{ln}\left({AA}^{*}\right)-\frac{q\left({\Phi }_{B}-E/n\right)}{kT}$$

where *T* is the temperature; *A*, the area; A*, the Richardson constant; *q*, the electronic charge of an electron; *n*, the ideal factor; and *k*, the Boltzmann constant. After the *J*-*E* characteristics of the device at various temperatures were measured as shown in Supplementary Fig. S2, the data were plotted according to the relationship log*J* vs. *E*^0.5^. As a result, straight lines (i.e., Schottky lines) were obtained as shown in Supplementary Fig. S3a. Then, the current densities in the absence of an electric field (*J*_0_) were determined by extrapolating the Schottky lines, as listed in Supplementary Table S1. Notably, the maximum *J*_0_ was two orders of magnitude greater than the minimum *J*_0_. Finally, the Richardson plot (ln*J*_0_/*T*^2^ − *T*^−1^) was drawn as shown in Supplementary Fig. S3b, and *Φ*_B_ was calculated from the slope of the Richardson line. Figure 3b shows the *Φ*_B_ values as functions of the fullerene-doping concentration. *Φ*_B_ for the non-doped (Au/TPA/Au) device was calculated to be 0.37 eV; this was almost same as *Φ*_B_ estimated from the work function of a Au electrode (~ 5.1 eV)^37^ and the ionization potential of TPA (~ 5.5 eV)^28^. The sigmoid-shaped behavior of *Φ*_B_ was observed for both C_60_- and C_70_-doped devices. However, C_70_ doping had a higher barrier-reduction effect than C_60_ doping. Remarkably, *Φ*_B_ for the Au/1 mol% C_70_-doped TPA/TPA/Au device was determined to be 0.021 eV; this satisfied the condition for ohmic contact (≤ 0.025 eV at room temperature). It is considered that there are multiple levels of barriers among the Au/fullerene-doped TPA/TPA. Although the details are unclear, the Richardson-Schottky plot analysis revealed that the barrier of the rate-determining step (probably hole injection at the Au/fullerene-doped TPA) is 0.021 eV in the Au/1 mol% C_70_-doped TPA/TPA/Au device. With the doping of more than 1 mol% C_60_ and C_70_, the barrier height did not decrease further, probably because it is difficult to dissolve C_60_ and C_70_ in organic solvents at a concentration of more than 1 mol% to TPA. Therefore, a technique for doping fullerenes at higher concentrations must be developed in the future. Overall, we reduced the hole injection barrier by 0.324 and 0.349 eV by introducing the C_60_- and C_70_-doped TPA layer as a hole injection layer, respectively, and successfully formed an ohmic contact at the Au/1 mol% C_70_-doped TPA interface.

### Consideration of hole-injection mechanism

We demonstrated barrier height reduction with hole injection at the Au/fullerene-doped TPA interface. Next, we focused on the barrier reduction mechanism. Lee reported that the hole-injection barrier was lowered by the interaction between the fullerene (C_60_ only) and the Al electrode^38^. In this study, we used fullerenes (both C_60_ and C_70_) as a dopant in the TPA layer. Therefore, the reduced hole-injection barrier was attributed to the intermolecular interaction between the fullerene and the TPA (and not the electrode). Actually, the ultraviolet photoelectron spectroscopy (UPS) result of the films of Au only and 1 mol% C_70_-coated Au shown in Supplementary Fig. S4 revealed that there is negligibly slight interaction between fullerene and Au electrode because the obtained both work functions were equivalent (~ 5.2 eV).

First, we conducted UV–vis spectrometry (V-650 spectrophotometer, JASCO Corp.) to observe the interaction, for example, the formation of a charge-transfer (CT) complex. However, no new absorption band appeared in the spectrum for a sample with a mixture of 1 mol% C_70_ and TPA, as shown in Supplementary Fig. S5. Considering the accuracy of the measuring instrument, it was suggested that negligibly slight amount of TPA-fullerene CT complex might be formed.

Next, photoelectron yield spectroscopy (PYS) was performed. As shown in Supplementary Fig. S6, the behaviors of PYS spectra for the films of TPA only and 1 mol% C_60_-doped TPA were similar, and the obtained both ionization potentials were also equivalent. Therefore, the fullerene dope would not form new energy levels associated with charge injection/extraction. This suggests that fullerenes contribute to barrier reduction as polarizable substances, not donors or acceptors.

Supplementary Fig. S7 shows the *J*-*E* characteristics of a Au/evaporated C_70_/TPA/Au layered device along with that of the Au/C_70_-doped TPA/TPA/Au layered device. Based on Supplementary Fig. S7b, the evaporated-C_70_/TPA and 1 mol% C_70_-doped TPA/TPA devices exhibited schottky-type (*J* ∝ *E*^0.5^) and ohmic-type (*J* ∝ *E*) current responses immediately after an electric field was applied, respectively, which indicates that both devices have different hole injection mechanisms. As a consequence, using the fullerene-doped TPA as a buffer layer has higher conductivity than using the evaporated C_70_. This suggests that it is important for fullerenes to penetrate the TPA layer morphologically.

**Figure 4:**
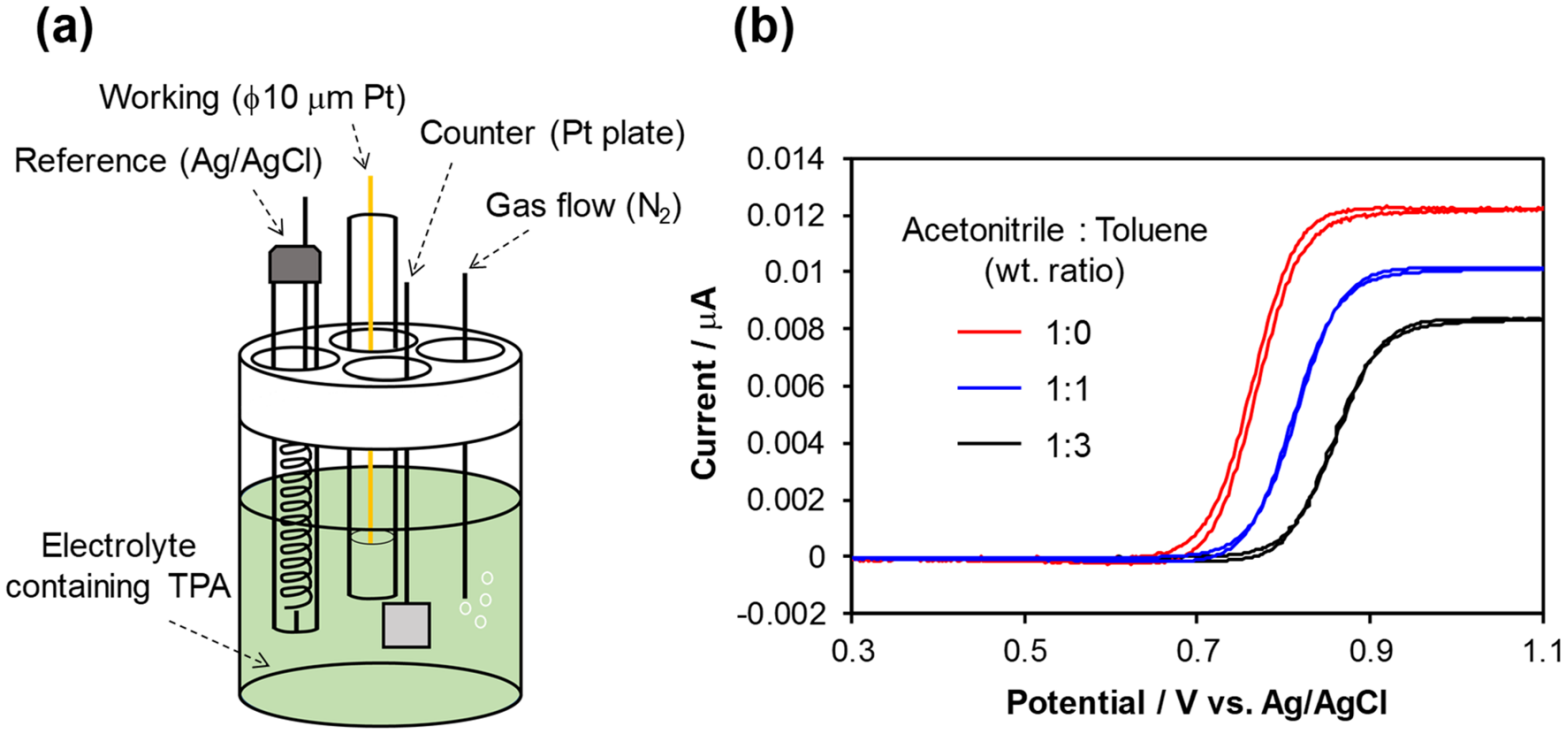
(**a**) Schematic diagram of the electrochemical cell used in this study. (**b**) Cyclic voltammograms of TPA at various acetonitrile:toluene weight ratios in the electrolyte at a supporting-electrolyte concentration of 50 mmol dm^−3^.

Then, we performed electrochemical analysis. Cyclic voltammetry was conducted using the electrochemical cell shown in Fig. 4a. Figure 4b shows cyclic voltammograms of TPA in mixed acetonitrile:toluene solutions with weight ratios of 1:0, 1:1, and 1:3. The half-wave potential (*E*_1/2_) for the oxidation of TPA (given by Eq. 2) was determined as the potential at which the current equals the half of diffusion limited current (*i*_d_)^39^.

2 $$\mathrm{TPA }\begin{array}{c}\stackrel{{k}_{f}}{\to }\\ \underset{{k}_{b}}{\leftarrow }\end{array} {\mathrm{TPA}}^{\cdot +}+{e}^{-}$$

As a result, *E*_1/2_ was obtained as 0.76 V, 0.81 V, and 0.86 V vs. Ag/AgCl for acetonitrile:toluene weight ratios of 1:0, 1:1, and 1:3, respectively. *i*_d_ differed depending on the acetonitrile:toluene weight ratios in the order of 1:3 < 1:1 < 1:0. These results indicate that TPA is more easily oxidized with a higher amount of acetonitrile. Because acetonitrile and toluene are polar and nonpolar solvents, respectively, their different ratios result in changes in the relative permittivity of the electrolytes. TPA was easily oxidized to TPA^+^ when it was surrounded by a polar solvent having a higher relative permittivity.

**Figure 5:**
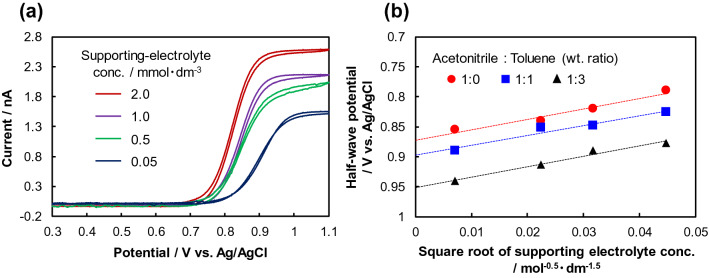
(**a**) Cyclic voltammograms of TPA at various supporting electrolyte concentrations with acetonitrile:toluene weight ratio of 1:1 in the electrolyte. (**b**) Dependence of half-wave potential of TPA oxidation on the concentration of the supporting electrolyte at various acetonitrile:toluene weight ratios in the electrolyte.

The mechanism in the solid-state device without a solvent was considered in light of the above findings. Because there are no ions in the solid phase state without any carrier injection, *E*_1/2_ must be calculated under the condition of the absence of a supporting electrolyte as an ion source. Figure 5a shows the cyclic voltammetry results for supporting electrolyte concentrations of 2.0, 1.0, 0.5, and 0.05 mmol dm^−3^ under the cell conditions shown in Fig. 4a. The oxidation potential of TPA decreased and *i*_d_ increased as the concentration of the supporting electrolyte increased. When *E*_1/2_ for various supporting electrolyte concentrations with various acetonitrile:toluene weight ratios in the electrolyte was calculated and plotted as a function of the square root of the supporting electrolyte concentration^40^, the linear function shown in Fig. 5b was obtained. Therefore, *E*_1/2_ without the supporting electrolyte can be calculated by extrapolating the straight line shown in Fig. 5b. Consequently, *E*_1/2_ for acetonitrile:toluene weight ratios of 1:0, 1:1, and 1:3 was 0.87 V, 0.89 V, and 0.94 V vs. Ag/AgCl, respectively. Even in the solid phase state, TPA was more likely to become TPA^+^ in the presence of a substance with a higher relative permittivity. Therefore, the ion–dipole interaction between TPA and fullerenes was suggested to facilitate hole injection from the Au electrode to TPA, because fullerenes have a relatively high dipole moment^41^. The relative permittivities of C_60_ and C_70_ were respectively measured to be ~ 3 and ~ 4 using an 879B LCR meter (B&K Precision Corp.) at 1 kHz. Because C_70_ has a higher relative permittivity than C_60_, the hole injection barrier was reduced more efficiently and an ohmic contact was probably formed with a larger ion–dipole interaction effect.

In the future, we plan to investigate the best doping material for reducing the barrier height from the viewpoint of relative permittivity.

Finally, electrochemical impedance spectroscopy was carried out to consider the interface energetic characteristics, and the result is shown in Supplementary Fig. S8. When the capacities at interfaces of Au/TPA and Au/fullerene-doped TPA were determined by fitting the impedance curves, the latter was larger than the former. This indicates that the depletion layer (energy barrier) formed at the Au/TPA interface becomes smaller due to the presence of fullerenes, and supports our proposed intermolecular ion–dipole interaction effect.

**Figure 6:**
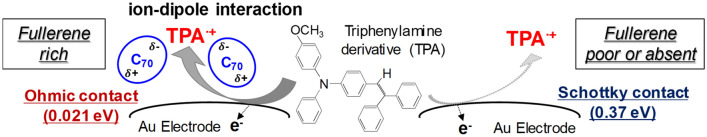
Schematic representation of the barrier height reduction mechanism with hole injection at the Au/fullerene-doped TPA interface.

Figure 6 summarizes the barrier height reduction mechanism with hole injection. Owing to the ion–dipole interaction between TPA and fullerenes, TPA is easily oxidized and stabilized to TPA^∙+^. This indicates that the rate constant of the forward reaction (*k*_f_) in Eq. (2) increased. Therefore, the hole injection at the Au/fullerene-doped TPA interface became highly efficient, because the equilibrium constant (*K*), expressed as the ratio of *k*_f_ and the rate constant of the reverse reaction (*k*_b_) in Eq. (2) (*K* = *k*_f_/*k*_b_), increased. Overall, the results suggested that the proposed hole injection technique has a novel mechanism. The UPS result (Fig. S4) revealed that there is a negligibly slight interaction between fullerene and Au electrode. The UV–vis result (Fig. S5) indicates that the TPA-fullerene CT complex is not formed probably. The PYS result (Fig. S6) evidences that the fullerene dope would not form new energy levels associated with hole injection. On the other hand, the contribution of intermolecular ion–dipole interaction (solvation effect) to the reduction of hole injection is strongly evidenced by the results of electrochemical analyses (Figs. 4 and 5). The ion–dipole interaction is involved to the ionization process of TPA. According to the Marcus theory^42,43^, the reorganization energy is involved in the activation energy and depends on the relative permittivity. In other words, the activation energy of TPA oxidation to TPA^∙+^ becomes low when the relative permittivity around TPA is high even in the solid and liquid states. This leads to a fact that the large ion–dipole interaction attributed to the high relative permittivity of medium contributes to the decrease in activation energy, which appeared in the *E*_1/2_ shift for TPA oxidation toward negative direction observed in Figs. 4 and 5. The decrease in activation energy can be applied to a solid state having a high relative permittivity such as fullerenes. Consequently, the ohmic contact at room temperature was successfully achieved at the Au/1 mol% C_70_-doped TPA interface (Fig. 3).

## Methods

### Materials

TPA, α-phenyl-4′-[(4-methoxyphenyl)phenylamino]stilbene, shown in Fig. 1a, was obtained from Ricoh Co. Ltd. Its purity was ensured to be one spot in thin-layer chromatography. C_60_ (≥ 99%) and C_70_ (≥ 97%) were obtained from Kanto Chemical Co., Inc. The reagents *o*-xylene (98.0+%), tetrahydrofuran (99.5+%), acetonitrile (99.8+%), and toluene (99.5+%) were purchased from Fujifilm Wako Pure Chemical Corp. Tetramethylammonium perchlorate (≥ 99.0%), TMAP, was purchased from Nacalai Tesque, Inc.

### Current density–electric field (*J*-*E*) measurement

After vacuum-depositing a 20-nm-thick Au electrode on a glass substrate using a VPC-260F instrument (ULVAC, Inc.), a 0.3-μm-thick fullerene-doped TPA layer was laminated by spin-coating a 10 wt% TPA-containing *o*-xylene solution at 3000 rpm for 30 s, in which fullerene was doped at concentrations of 0.01, 0.1, 0.5, 1, 2 (only for C_60_), and 5 (only for C_70_) mol% to TPA. The thickness of 0.3 μm of the fullerene-doped TPA layer was strategically employed in order to laminate the layer completely by a cast-coat method. Possibly, the 0.3-μm thichness of the layer influences the hole-transport resistance in the device. However, the influence should be negligible in this study because this work focuses on hole-injection property at the Au/fullerene-doped TPA interface. Then, a 50 wt% TPA-containing tetrahydrofuran (THF) supersaturated solution was spin-coated at 3000 rpm for 30 s onto the fullerene-doped TPA layer to obtain a 5.5-μm-thick TPA layer. The thickness of the fullerene-doped TPA and TPA layers was determined using a Surfcom 130A contact-type thickness meter (Tokyo Seimitsu Co., Ltd.). The supersaturated TPA solution was used so that the lower layer was not dissolved. Also, we used THF as a solvent because fullerenes are almost insoluble in THF. As a result, the complete TPA-containing double layers were obtained as shown in Supplementary Fig. S9. A counter Au electrode was finally vacuum-deposited in the same manner to fabricate the stacked Au/fullerene-doped TPA/TPA/Au device shown in Fig. 1b. The *J*-*E* characteristics of the fabricated devices were measured at various temperatures at 1 × 10^–3^ Pa under dark conditions in a vacuum chamber by using a source meter (Keithley 2612A).

### Cyclic voltammetry

A Pt disk with a diameter of 10 μm, a Pt plate, and a Ag/AgCl/saturated KCl were used as working, counter, and reference electrodes, respectively. The ϕ10 μm Pt electrode was prepared using the procedure described in a previous study^31^. Two types of electrolyte were used. One consisted of 5.0 mmol dm^−3^ TPA; a supporting electrolyte of 50 mmol dm^−3^ TMAP; and acetonitrile/toluene mixed solvent in weight ratios of 1:0, 1:1, and 1:3. The other consisted of 5.0 mmol dm^−3^ TPA; TMAP in concentrations of 2.0, 1.0, 0.5, and 0.05 mmol dm^−3^; and acetonitrile/toluene mixed solvent in weight ratios of 1:0, 1:1, and 1:3. Cyclic voltammetry was conducted at a scan rate of 50 mV s^−1^ in the potential range of 0.3–1.1 V vs. Ag/AgCl using the electrochemical cell shown in Fig. 4a and a HA-150 potentiostat (Hokuto Denko Corp.). Degassing by N_2_ gas bubbling was performed before measurements.

## Supplementary Information

Supplementary Figures.
