# Supplementary material for: Highly efficient hole injection from Au electrode to fullerene-doped triphenylamine derivative layer
Source: Sci Rep. 2022 May 4;12:7294. doi: 10.1038/s41598-022-10983-6 (PMC9068712; doi:10.1038/s41598-022-10983-6)
Supplement: Supplementary file 1 — Supplementary Figures. [file 41598_2022_10983_MOESM1_ESM.docx]

Supplementary Information for

**Highly efficient hole injection from Au electrode to fullerene-doped triphenylamine derivative layer**

Shofu Matsuda, Chikara Itagaki, Kyoya Tatsuguchi, Masamichi Ito, Hiroto Sasaki, Minoru Umeda *

*Department of Materials Science and Technology, Graduate School of Engineering, Nagaoka University of Technology, 1603-1 Kamitomioka, Nagaoka, Niigata 940-2188, Japan*

^*^ Corresponding author: mumeda@vos.nagaokaut.ac.jp (M. Umeda)

**Figure S1.** *J*-*E* characteristics of the Au/1 mol% C_70_-doped TPA/TPA/Au layered device at the temperatures of 4.5°C and -22.3°C plotted on a log-log scale.

S1

**Figure S2.** *J*-*E* characteristics of the Au/0.01 mol% C_70_-doped TPA/TPA/Au layered device at various temperatures.

**Figure S3.** (a) Schottky and (b) Richardson plots of the Au/0.01 mol% C_70_-doped TPA/TPA/Au layered device.

**Table S1**. *J*_0_ values obtained from Schottky line (Figure S3a) at each temperature.

| Temperature (*T*) / °C | Current density at *E* = 0 (*J_0_*) / µA cm^-2^ |
| --- | --- |
| 27.8 | 1.3×10^-4^ |
| 18.1 | 4.5×10^-5^ |
| -26.1 | 5.5×10^-5^ |
| -43.4 | 1.3×10^-6^ |

S2

**Figure S4.** UPS spectra for the films of Au only (black) and 1 mol% C_70_-coated Au (red). The 1 mol% C_70_-coated Au film was fabricated by spin-coating 1 mol% C_70_-containing *o*-xylene solution without TPA on Au. The ultraviolet photoelectron spectroscopy (UPS) was performed with a Nexsa instrument (Thermo Fisher Scientific K.K.). A He lamp (He(I): 21.22 eV) was used as an UV light source. According to the literature (C. D. Frisbie et al., *J. Am. Chem. Soc.* **2011**, *133*, 19864.), the work functions of Au only and 1 mol% C_70_-coated Au films were calculated to be 5.22 eV and 5.17 eV, respectively. The accuracy of the measuring instrument is $\pm$0.05 eV.

**Figure S5.** UV-vis spectra for the *o*-xylene solution samples of TPA only, C_70_ only, and mixture of 1 mol% C_70_ and TPA. The accuracy of the measuring instrument is $\pm$0.1 nm.

S3

**Figure S6.** PYS spectra for the films of (a) TPA only and (b) 1 mol% C_60_-doped TPA. The photoelectron yield spectroscopy (PYS) was performed with a BIP-M25 instrument (Bunkoukeiki Co., Ltd.). The PYS measurement was conducted in the wavelength range of 200-400 nm at a voltage of 100 V under atmospheric pressure. The light-irradiation area was 2×2 mm^2^. According to the literature (Y. Nakayama et al. *Appl. Phys. Lett.* **2008**, *93*, 173305.), the ionization potentials of TPA and fullerene-doped TPA were determined to be 5.23 eV and 5.25 eV, respectively. The accuracy of the measuring instrument is $\pm$0.05 eV.

**Figure S7.** *J*-*E* characteristics of the Au/evaporated C_70_/TPA/Au (blue) and the Au/1 mol% C_70_-doped TPA/TPA/Au (red) layered devices plotted on (a) a linear scale and (b) a log-log scale. The thickness of evaporated C_70_ layer was determined as 0.3 μm using a Surfcom 130A contact-type thickness meter, which is equivalent to the thickness of C_70_-doped TPA layer.

S4

**Figure S8.** Nyquist plots for the Au/TPA/Au (black) and the Au/1 mol% C_60_-doped TPA/Au (red) layered samples. The electrochemical impedance spectroscopy (EIS) was performed with a PAR283 potentiostat (EG&G) and a Solartron1260 frequency response analyzer (Solartron Analytical). The EIS measurement was conducted at a DC voltage of 0 V in a frequency range between 1 MHz and 1 Hz with a sine wave AC amplitude of 100 mV. The electrode surface area was 4.0 mm^2^. The thickness of TPA and fullerene-doped TPA layers were 0.75 μm. The interfacial capacities at Au/TPA and Au/fullerene-doped TPA were determined to be 0.04 nF and 0.11 nF, respectively, by fitting their impedance curves.

**Figure S9.** The cross-section SEM images of the glass substrate/Au/1 mol% C_70_-doped TPA/TPA layered sample. The image was obtained using a Hitachi SU8230 field emission scanning electron microscope (FE-SEM) at an acceleration voltage of 5.0 kV at a mignification of ×70 k.

S5
